# Feasibility and efficacy of a psychological graded early warning and intervention system for hospitalised patients in a general hospital

**DOI:** 10.3389/fmed.2026.1670946

**Published:** 2026-01-30

**Authors:** Yanping Duan, Wenqi Geng, Boheng Zhu, Lili Shi, Jinya Cao, Tao Li, Ruixue Sun, Weixuan Qu, Chunfeng Xiao, Yinghan Xie, Jiarui Li, Jianhua Du, Jiaojiao Hu, Yinan Jiang, Jing Wei

**Affiliations:** Department of Psychological Medicine, Peking Union Medical College Hospital, Chinese Academy of Medical Sciences and Peking Union Medical College, Beijing, China

**Keywords:** consultation liaison psychiatry, general hospital, graded early warning intervention, patient safety, psychological assessment

## Abstract

**Objective:**

To evaluate the feasibility and effect of a psychological graded early warning and intervention system for hospitalised patients in a general hospital.

**Methods:**

The psychological screening of patients within 48 h of hospitalisation was conducted using the Union Physio-Psycho-Social Assessment Questionnaire (UPPSAQ), the Life Events Scale (LES), the Hospitalized Patients’ Expectations for Treatment Scale-patient version (HOPE-P), and the Patient–Doctor-Relationship Questionnaire (PDRQ). Based on the obtained results, the warning level was divided into a recommendation level, an intervention level and a report level, after which a liaison consultation-based psychiatric intervention was administered. Patients who completed the assessments were invited for retest after 3 months.

**Results:**

A total of 1757 patients completed the psychological assessment upon admission, and 338 of them completed the follow-up psychological assessment after three months. Only 4 patients (1.2%) scored at the normal level, 199 patients (58.9%) scored at the recommendation level (grade = 1), 125 patients (37.0%) scored at the intervention level (grade = 2), and 10 patients (3.0%) scored at the report level (grade = 3). The consultation rate was 7.5% for grade 1, 19.2% for grade 2, and 70% for grade 3. After the liaison consultation intervention, the total UPPSAQ score at grades 2 and 3 significantly improved (*t* = −2.110, *p* = 0.037), the HOPE-P score was lower than that at admission (*t* = 0.478, *p* < 0.001), and no significant changes were noted in the PDRQ or LES scores (*p* > 0.05).

**Conclusion:**

It is feasible to conduct comprehensive psychological screenings of patients hospitalised in general hospitals to implement early warning-based graded interventions. Implementing the liaison consultation intervention according to the graded early warning system can improve the psychosomatic health of patients.

**Clinical trial registration:**

http://www.chictr.org.cn/, identifier ChiCTR2300075262.

## 1 Introduction

The first-ever World Patient Safety Day was held on September 17, 2019. This day signifies that patient safety constitutes a core aspect of public health, emphasising that “addressing systemic, organisational, cultural and behavioural drivers of patient harm remains extremely challenging and many known problems remain unsolved.” While treating acute illnesses, care systems are obliged to provide optimal and standardised treatment for all patients at every level to avert any further harm (1). The World Health Organization (WHO) proposed that mental health should be accorded a prominent position on global development and humanitarian agendas (2, 3) and advocated that “There can be no true health without mental health.” The psychological and behavioural issues of patients and medical staff are crucial elements that impact patient safety management (4, 5).

How to effectively integrate mental health into patient safety practices in general hospitals remains an unresolved challenge. Conducting one-on-one psychological interviews in general hospitals is not feasible. However, it is possible to utilise psychological self-assessment to screen the mental health status of patients and subsequently conduct graded interventions on the basis of psychological evaluation (6). A previous study conducted expert consultation in which psychological safety factors such as suicide or self-injury; treatment compliance; adverse health behaviours; depression, anxiety, or other common psychological problems; and doctor–patient relationship issues were incorporated into the patient safety management system (4). The graded intervention of patients with mental health early warning systems may improve the safe management of the mental health of such patients in general hospitals (7). The psychological health status of patients with physical diseases in general hospitals encompasses multiple dimensions, such as physical pain, emotions, and impaired social functions. A comprehensive assessment scale that considers the three dimensions of physiology, psychology, and society is necessary to reduce content overlap, time consumption, and decreased patient engagement. Therefore, we chose the original and mature Union Physio-Psycho-Social Assessment Questionnaire (UPPSAQ), which is suitable for the Chinese context, to screen for psychological and behavioural conditions (8).

Consultation–liaison psychiatry (CLP) has developed rapidly, and the liaison consultation model has progressively supplanted the invited consultation model, which is more proactive. Psychiatric consultation at an early stage is associated with fewer 30-day and 7-day readmissions (9). It is practical to implement a three-stage ward round and follow-up system for patients with somatic diseases in general hospitals (10, 11). Through patients’ self-psychological assessments, potential mental health problems can be screened, psychological problems can be graded, and then CLP intervention can be administered. In this study, early assessments and warnings as well as graded-based interventions were implemented for the mental health conditions of inpatients in general hospitals, and the feasibility and effectiveness of this process was evaluated.

## 2 Methods

This study was a part of the Capital Health Development Research Project “Establishment of a system based on psychological behaviour assessment and graded early-warning intervention to promote medical quality and patient safety in general hospitals.” In this project, 180 patients from each of the 10 departments of Gynaecology, Urology, Orthopaedics, General surgery, Respiratory Medicine, Immunology, Endocrinology, Neurology, Gastroenterology and Cardiology were enrolled for psychological behaviour assessment and graded early warning, and CLP interventions were administered. A reassessment of psychological behaviour was conducted 3 months after the patients were discharged.

### 2.1 Participants

From July 4, 2022, to April 26, 2024, patients in the wards of Gynaecology, Urology, Orthopaedics, General surgery, Respiratory Medicine, Immunology, Endocrinology, Neurology, Gastroenterology and Cardiology received psychological screening.

The inclusion criteria were inpatients who had been hospitalised for more than 24 h and who were 18 years or older.

The exclusion criteria included inpatients who had restricted language proficiency or intellectual disability, visual or auditory impairments, or other factors that could prevent them from providing informed consent or completing the assessments.

### 2.2 Data collection

The Union Physio-Psycho-Social Assessment Questionnaire (UPPSAQ), Life Events Scale (LES), Hospitalized Patients’ Expectations for Treatment Scale-patient version (HOPE-P), and Patient–Doctor-Relationship Questionnaire (PDRQ) were chosen to assess the patients’ social, psychological and physiological conditions. The patients were invited to undergo retest using these scales after 3 months.

UPPSAQ: The UPPSAQ is an effective scale for evaluating people’s overall health status and is applicable in general hospitals. The 8-factor model of this scale encompasses physical discomfort and anxiety, sleep, pain, hypochondria, emotion, sexual function and feeling discomfort, happiness and satisfaction, and social disorders (8). The scoring criteria are such that a factor average score greater than 1 denoted a high factor index, and the total average score is 3 points. A total score above 65 indicates possible psychosomatic health problems, whereas a score above 100 indicates significant psychosomatic problems (12).

LES: The LES demonstrates good convergent validity and discriminant validity among patients in general hospitals. The LES is positively correlated with depression, loneliness and hopelessness; negative life events had a correlation with quality of life, family function, and social support, both in cases of suicide and among living controls (13).

HOPE-P: The HOPE-P is a reliable and valid assessment instrument for gauging the expectations of inpatients in general hospitals. HOPE-P has three dimensions: doctor–patient communication expectations, treatment outcome expectations, and disease management expectancy (14).

PDRQ: The PDRQ demonstrates good internal consistency. A two-factor model of relationship quality and treatment quality was proposed. The Chinese version of the PDRQ is a valid and reliable rating scale that can assess doctor–patient relationships among Chinese patients (15).

### 2.3 Psychological graded early-warning and intervention system

The grading of the total UPPSAQ score was as follows: a score of 0–64 was considered the normal level, 65–99 was the recommendation level, and a score exceeding 100 was categorised as the intervention level. For the suicide item of the UPPSAQ, a score of 1 was classified as the intervention level, and a score of 2 was classified as the reporting level. Based on the total LES score, a score ranging from 23 to 31 was designated as the recommendation level and 32 points and above were considered the intervention level. The grades were determined based on a comparison between the higher intervention level determined by the UPPSAQ and LES, and ultimately, they were divided into 4 levels: grade 0 as normal, grade 1 as the recommendation level, grade 2 as the intervention level, and grade 3 as the reporting level (detailed in Table 1).

**Table 1:** Psychological graded early warning and intervention system.

Grade	Intervention level	UPPSAQ	LES	Suicide	Meaning of intervention
Grade 0	Normal level	0–64	0–23	0	No indication for CLP intervention
Grade 1	Recommendation level	65–99	23–31	0	The doctors in charge proposed CLP consultation according to the needs of the inpatients.
Grade 2	Intervention level	≥100	≥32	1	The CLP working group raised the patient’s possible psychosomatic problems to the wards and asked for the patient’s consent before proposing the CLP consultation needs.
Grade 3	Reporting level	-	-	2	The CLP working group proactively contacted the ward and examined the patient, informed the suicide intervention process, and reported the high risk of suicide to the hospital medical service.

Hierarchical intervention procedures (detailed in Table 1):

Garde 1: The doctor in charge of the ward and the patient himself or herself reviewed the evaluation results and independently determined whether CLP consultations were necessary. For the inpatients who received consultations, follow-up was performed using the inpatient treatment approach.
Grade 2: The CLP working group raised the patient’s possible psychosomatic problems to the wards and asked for the patient’s consent before the CLP consultation needs were proposed. For the inpatients who received consultations, follow-up was conducted using the inpatient treatment approach.
Grade 3: The CLP working group proactively contacted the ward and examined the inpatients who met grade 3 criteria, informed the ward and inpatient guardian of the suicide intervention process, and reported a high risk of suicide to the hospital medical service.

### 2.4 Statistical analysis

Descriptive statistics: Continuous variables are described as the means ± standard deviations (means ± SDs) if the data conformed to a normal distribution and as the medians (minimums, maximums) if the data were not normally distributed. For categorical variables, the counts and percentages are presented. Statistical analysis: Independent sample *t* tests and paired sample *t* tests were employed for normally distributed data, and nonparametric tests (Wilcoxon signed-rank tests) were used for nonnormally distributed data. The significance level was set as *p* < 0.05.

## 3 Results

A total of 1757 patients completed the psychological assessment upon admission, and 338 of these patients completed the follow-up psychological assessment after three months. There were 1,418 patients who refused the 3-month follow-up. A comparison of the demographic characteristics of the follow-up patients and those who refused follow-up revealed that the patients in the refused follow-up group were older (t = 4.742, *p* < 0.001), more commonly widowed (*x^2^* = 15.506, *p* = 0.004), less commonly employed (*x^2^* = 24.563, p < 0.001), and had a lower educational level (*x^2^* = 15.360, *p* = 0.002). Details of the demographic information of the patients are shown in Table 2.

**Table 2:** Demographic information of the inpatients in the follow-up group and refused follow-up group.

Item	Follow-up group (*n* = 338)		Refused follow-up group (*n* = 1,418)		*t/x^2^*	*p*
Sex					0.335	0.563
Male	145	42.9%	633	44.6%		
Female	193	57.1%	785	55.4%		
Age	46.68 ± 15.00		51.23 ± 16.05		4.742	**<0.001**
Nationality					0.273	0.601
Han	318	94.1%	1,323	93.3%		
Others	20	5.9%	95	6.7%		
Residence					0.786	0.375
Urban	262	77.5%	1,130	79.7%		
Rural	76	22.5%	288	20.3%		
Marital status					15.506	**0.004**
Married	256	75.7%	1,117	78.8%		
Single	55	16.3%	207	14.6%		
Divorced	12	3.7%	44	3.2%		
Widowed	2	0.6%	30	2.2%		
Others	12	3.7%	15	1.2%		
Employment status					24.563	**<0.001**
Employed	154	45.6%	555	39.1%		
Student	35	10.4%	80	5.6%		
Retired	72	21.3%	442	31.3%		
Housewife	12	3.6%	79	5.6%		
Unemployed	38	11.1%	172	12.1%		
Others	27	8.0%	90	6.3%		
Education					15.360	**0.002**
Primary school	13	3.8%	107	7.5%		
Junior high school	52	15.4%	279	19.7%		
Senior high school	65	19.3%	314	22.1%		
University and above	208	61.5%	718	50.6%		

*p*<0.05.

Among these patients, only 4 (1.2%) were classified as grade 0. There were 199 patients (58.9%) at grade 1, 125 patients (37.0%) at grade 2, and 10 patients (3.0%) at grade 3. Among the patients in grade 1, 15 patients (7.5%) received a CLP consultation. Compared with the scores at admission, the UPPSAQ total scores tended to increase after 3 months (t = −5.128, *p* < 0.001). For the patients at grade 2, the rate of CLP consultation was 19.2%, and no substantial difference was detected in the UPPSAQ score between admission and after 3 months. Among the grade 3 patients, seven patients (70%) received a CLP consultation. The total UPPSAQ score decreased (t = 2.576, *p* = 0.030) (detailed in Table 3).

**Table 3:** The effectiveness and feasibility of the psychological graded early warning and intervention system.

Grade	CLP rate (person-time)	UPPSAQ At admission	UPPSAQ After 3 months	*t* score	95% CI	*p* score
Grade 0 (*n* = 4)	0	11 (10, 48)	27.00 (6.00, 45.00)	-		-
Grade 1 (*n* = 199)	15 (7.5%)	51.00 ± 19.77	61.81 ± 21.89	−5.128	−12.398-5.511	**0.000**
Grade 2 (*n* = 125)	24 (19.2%)	72.65 ± 24.62	69.95 ± 25.39	1.334	−1.304-6.696	0.185
Grade 3 (*n* = 10)	7 (70%)	105.10 ± 34.44	80.40 ± 34.89	2.576	3.010–46.390	**0.030**

*p*<0.05.

We analysed alterations in psychosomatic health, treatment expectations, and doctor–patient relationships among grade 2 or grade 3 patients. The findings from the follow-up evaluations revealed a decrease in the total UPPSAQ score, such as for physical discomfort and anxiety and social disturbance and depression (*p* < 0.05). Concurrently, the happiness and satisfaction index improved (*t* = −2.656, *p* = 0.008). The overall HOPE-P score also decreased (*t* = 4.718, *p* < 0.005). By contrast, no significant difference in the LES or PDRQ was detected between admission and after 3 months (*p* > 0.05) (Table 4).

**Table 4:** Analysis of the mental health conditions of grade 2 or grade 3 inpatients at admission and after 3 months.

Item	Scores^a^	Scores^b^	*t*/*z*	*p*
UPPSAQ	75.05 ± 26.71	70.72 ± 26.19	2.110	**0.037**
Somatic discomfort and anxiety	0.90 (0.20, 2.80)	0.90 (0.10, 2.40)	−2.132	**0.033**
Sleep	0.80 (0, 3.00)	0.90 (0, 3.00)	−0.022	0.983
Pain	0.70 (0, 3.00)	0.30 (0, 3.00)	−1.482	0.138
Discomfort with sexual function	2.00 (0, 3.00)	2.00 (0, 3.00)	−0.588	0.557
Happiness and satisfaction	1.60 (0, 3.00)	1.80 (0, 3.00)	−2.656	**0.008**
Social dysfunction	0.70 (0, 2.70)	0.50 (0, 2.70)	3.500	**0.000**
Depressive emotion	0.90 (0, 2.90)	0.80 (0, 2.80)	−2.640	**0.008**
Hypochondriasis	1.00 (0, 2.80)	0.90 (0, 3.00)	−1.518	0.129
LES	6 (0, 527)	8.5 (0, 278)	−1.104	0.269
Positive life events	0 (0, 228)	0 (0, 158)	−0.796	0.426
Negative life events	1 (0, 464)	2 (0, 251)	−1.638	0.101
Family life events	4 (0, 311)	6 (0, 240)	−0.886	0.376
Work or study life events	0 (0, 200)	0 (0, 96)	−0.443	0.665
Social and other life events	0 (0, 80)	0 (0, 70)	−1.481	0.139
HOPE-P	31.58 ± 5.90	27.90 ± 4.94	4.718	**0.000**
Doctor–patient relationship	9.98 ± 2.40	10.51 ± 1.89	−1.883	0.063
treatment outcome	18.51 ± 3.17	16.77 ± 4.18	3.330	**0.001**
Disease management attitude	4.00 (0, 4.00)	0 (0, 4.00)	−7.929	**0.000**
PDRQ	32.55 ± 4.87	32.37 ± 5.45	0.262	0.794

^a^At admission; ^b^After 3 months. *p*<0.05.

## 4 Discussion

In this study, inpatients hospitalised in a general hospital commonly exhibited various mental health issues. The psychological graded early warning and intervention system revealed approximately one-third of the inpatients might require CLP intervention, and 2.9% might have suicide risk. The CLP intervention has the potential to improve the psychosomatic health of hospitalised patients.

Common mental health problems among inpatients include affective disorders (16), anxiety disorders (17), alcohol/substance abuse (16, 17), schizophrenia, personality disorders and intellectual disability (18), and cognitive problems and organic psychiatric disorders (19). Patients hospitalised in general hospitals are at an increased risk of engaging in violent or suicidal behaviour (16). A previous study reported that 45% of inpatients were prescribed psychotropic medications, with 17.7% on antidepressants, 11% on antipsychotics, 19% on benzodiazepines, 3% on mood stabilisers, 2% on cholinesterase inhibitors, 0.5% on antiparkinsonian medication, and 1% on zolpidem and melatonin (19). Our previous study revealed that the mental disorders of inpatients in a general hospital could cover all mental diseases (11). In this study, only 1.2% of the inpatients were in a normal mental state, suggesting that almost 98.8% of the inpatients might have had psychosomatic health problems. Among the grade 1 patients, the liaison consultation psychiatrist accepted the hospital’s consultation invitation passively. The liaison consultation psychiatrist actively provided the psychiatric consultation to the grade 2 patients. In addition to active consultation, the suicide risk of the grade 3 patients was also addressed. The results indicated that active consultation interventions might be helpful for improving the mental health of inpatients in general hospitals.

The mental health status of the hospiatlised grade 1 patients was more severe at the 3-month follow-up than at admission, but the overall average score was lower than that of the grade 2 patients. The mental health status of the hospitalised grade 3 patients improved significantly at the 3-month follow-up compared with that at admission, but the overall average score was still higher than that of the grade 2 patients. The explanation for this issue is rather complex and may be related to various factors, such as patients at the reporting level receiving more attention from medical staff, the participation of family members in 24-h care enhancing social support, and drugs or psychological treatment possibly alleviating patients’ psychological problems. Owing to the low follow-up rate, this may have affected the follow-up results. Compared with the patients in the follow-up group, the patients in the follow-up refusal group were older, more commonly widowed, and had lower educational levels and occupational statuses, which may have affected their views and acceptance of mental health; thus, the importance given to the mental health problems of these patients is insufficient.

Suicide risk is a major concern among hospitalised patients in general hospitals. Hospitalised patients with both physical and mental disorders, such as cancer, epilepsy, chronic obstructive pulmonary disease, asthma, stroke, and chronic pain conditions, might be more likely to experience suicidal thoughts (20, 21). Patients in the emergency department (22) and adolescents (23) exhibit a higher suicide risk than the general population does. The probability of completed suicide is significantly greater among those who had expressed suicidal ideation than among those who had not (24). A total of 88% of general hospital suicide victims had one or more diagnoses of Axis I psychiatric disorders (25). Major depressive disorder is the most prevalent disorder and is more commonly seen in general hospitals (25). Psychiatric inpatients who engage in suicidal behaviour are typically younger women with psychiatric diagnoses and a history of previous suicide attempts (26). By contrast, non-psychiatric suicidal inpatients communicate less about suicide, carry out suicidal acts more quickly after admission, and use more violent suicidal methods (26). Suicidal acts occurring outside the hospital, the use of violent suicidal methods, and male sex all increase the risk of suicide mortality among suicidal inpatients (26). In this study, approximately 3% of the hospitalised patients in the survey reported frequent suicidal thoughts, and their comprehensive mental health status clearly worsened.

Psychological screening and psychological intervention hold the potential to curtail suicidal behaviours. Previous studies have shown that 20.9% of patients have made at least one suicide attempt in the emergency department (22). Boudeaux et al. carried out the “Emergency Department Safety Assessment and Follow-up Evaluation Screening Outcome Evaluation” to investigate the feasibility and efficacy of universal suicide risk screening in enhancing the detection of suicide risk within the emergency department. A three-phase interrupted time series design was used: the first phase involved treatment as usual, the second phase involved universal screening, and the third phase involved a treatment component for universal screening. Across the three phases, the documented screening rate increased from 26% (Phase 1) to 84% (Phase 3) (27). Moreover, universal screening was not found to be an excessive burden (28). In this study, patients who were psychologically screened for suicide risk received more CLP interventions and experienced a recovery of mental health indicators during the subsequent 3 months of follow-up, but the scores for psychosomatic health were still significantly higher than those for other inpatients; thus, these patients need to be included in longer-term outpatient follow-up interventions.

However, there are still deficiencies in the identification and intervention of psychological problems among patients in general hospitals. First, the proficiency of medical staff in detecting mental disorders within the somatic wards of general hospitals is insufficient (29, 30). Second, the screening instruments used were relatively rudimentary, with the current study primarily centred on anxiety and depression. Stigmatisation of mental health is prevalent within general hospital settings and has a negative influence on the quality of patient care (31). Simply assessing a patient’s psychiatric symptoms might increase the patient’s stigma (32, 33), and intentionally withholding or refusing to answer might also increase the coverage and accuracy of assessment screening. The stigma of medical staff could also affect the recognition of patients’ mental health problems (34). However, some studies have focused on patients’ psychological and physical symptoms, quality of life, and life satisfaction (35). Third, liaison consultations are becoming more popular than invitational consultations are (36). Planning should ideally be integrated into hospitals’ internal decision-making procedures to ensure that the emphasis on service policy is “bottom-up” rather than “top-down” (37). In clinical practice, comprehensive and standardised screening and intervention models are gradually evolving (38). The “Proactive Integrated Psychological Medicine” initiative in the UK represents a combination of proactive consultation and integrated care, as detailed in a service manual that has been field tested (39). In this study, the liaison consultation professional team collaborated with the ward medical teams to jointly identify and intervene in the mental health status of inpatients. As the warning level increased, the consultation rate progressively increased.

This study had several limitations. First, a low follow-up rate was observed. Despite the efforts of the staff, who contacted the patients via phone to conduct the assessment and provided at least three reminders, the rate remained suboptimal. Potential reasons included the weak subjective willingness of some patients to motivate themselves postdischarge, the stigma experienced by patients and their families, the unfortunate deaths of some patients with serious somatic illness after discharge and the individual’s ability to use the network, all of which had an impact on the follow-up rate. Second, owing to the limited timing of the intervention and the duration of follow-up, it was difficult to capture the detailed temporal changes and effects. Future studies should strengthen the follow-up timing and duration. Third, psychological and behavioural health problems had worsened after 3 months compared to at admission in the grade 1 patients. Psychiatrists did not actively offer consultations to these patients, instead, the consultation request was initiated by the patient or the ward doctor. It might also be necessary for active psychiatrists to visit inpatients with a low risk of psychological problems. It is also possible that patients with combined mental and psychological problems might receive more attention from ward doctors and nurses. The grade 1 patients might not have received extra attention from medical staff. The patient’s condition, length of hospital stay, and other social and psychological factors could affect the patient’s mental health status. Fourth, the consultation rate was inadequate, which might be attributed to rapid patient turnover within the ward. In this study, dedicated personnel were assigned to periodically review the evaluation results to ensure timely intervention to the greatest extent possible. Future research should consider leveraging information technology to enhance the dissemination of psychological evaluation results and thereby improve the effectiveness of interventions.

## 5 Conclusion

Inpatients hospitalised in general hospitals have a high demand for CLP intervention. It is feasible to conduct comprehensive psychological screenings of patients hospitalised in general hospitals to implement early warning-graded interventions. Liaison consultation intervention according to the graded early warning system can improve the psychosomatic health of patients. Owing to the low follow-up rate, which may affect the results of the graded psychological early warning system and intervention, during the CLP intervention, greater attention should be given to the identification of the mental health status of elderly, low-educated or socially unsupported hospitalised patients and the implementation of customised interventions for them.

## Data Availability

The original contributions presented in the study are included in the article/supplementary material, further inquiries can be directed to the corresponding authors.
